# Nanoliposomes for encapsulation and delivery of the potential antitumoral methyl 6-methoxy-3-(4-methoxyphenyl)-1*H*-indole-2-carboxylate

**DOI:** 10.1186/1556-276X-6-482

**Published:** 2011-08-03

**Authors:** Ana S Abreu, Elisabete MS Castanheira, Maria-João RP Queiroz, Paula MT Ferreira, Luís A Vale-Silva, Eugénia Pinto

**Affiliations:** 1 Centre of Physics (CFUM), University of Minho, Campus de Gualtar, 4710-057 Braga, Portugal; 2 Centre of Chemistry (CQ/UM), University of Minho, Campus de Gualtar, 4710-057 Braga, Portugal; 3 Laboratory of Microbiology, Faculty of Pharmacy and Centre of Medicinal Chemistry (CEQUIMED), University of Porto, Rua Aníbal Cunha 164, 4050-047 Porto, Portugal

## Abstract

A potential antitumoral fluorescent indole derivative, methyl 6-methoxy-3-(4-methoxyphenyl)-1*H*-indole-2-carboxylate, was evaluated for the *in vitro *cell growth inhibition on three human tumor cell lines, MCF-7 (breast adenocarcinoma), A375-C5 (melanoma), and NCI-H460 (non-small cell lung cancer), after a continuous exposure of 48 h, exhibiting very low GI_50 _values for all the cell lines tested (0.25 to 0.33 μM). This compound was encapsulated in different nanosized liposome formulations, containing egg lecithin (Egg-PC), dipalmitoyl phosphatidylcholine (DPPC), dipalmitoyl phosphatidylglycerol (DPPG), DSPC, cholesterol, dihexadecyl phosphate, and DSPE-PEG. Dynamic light scattering measurements showed that nanoliposomes with the encapsulated compound are generally monodisperse and with hydrodynamic diameters lower than 120 nm, good stability and zeta potential values lower than -18 mV. Dialysis experiments allowed to monitor compound diffusion through the lipid membrane, from DPPC/DPPG donor liposomes to NBD-labelled lipid/DPPC/DPPG acceptor liposomes.

## Introduction

Anticancer drugs are crucial agents in the global approach to fight cancer. Drug-loaded nanoparticles provide a perfect solution to afford higher therapeutic efficacy and/or reducing toxicity and the possibility of targeting cancer tissues. Nanoliposomes are one of the best drug delivery systems for low molecular weight drugs, imaging agents, peptides, proteins, and nucleic acids. Nanoliposomes are able to enhance the performance of bioactive agents by improving their bioavailability, *in vitro *and *in vivo *stability, as well as preventing their unwanted interactions with other molecules [1-3]. It is believed that the efficient antitumor activity can be attributed to the selective delivery and the preferential accumulation of the liposome nanocarrier in the tumor tissue via the enhanced permeability and retention effect [4-6].

Nanoliposomes may contain, in addition to phospholipids, other molecules such as cholesterol (Ch) which is an important component of most natural membranes. The incorporation of Ch can increase stability by modulating the fluidity of the lipid bilayer preventing crystallization of the phospholipid acyl chains and providing steric hindrance to their movement. Further advances in liposome research found that surface modification with polyethylene glycol (PEG), which is inert in the body, generally reduces the clearance of liposome by RES, and therefore allows longer circulatory life of the drug delivery system in the blood [3]. Pegylated liposomal doxorubicin has shown great prolonged circulation and substantial efficacy in breast cancer treatment [7]. The net charge of nanoliposomes is also an important factor and generally anionic and neutral liposomes survive longer than cationic liposomes in the blood circulation after intravenous injection [8,9].

**Figure 1:**
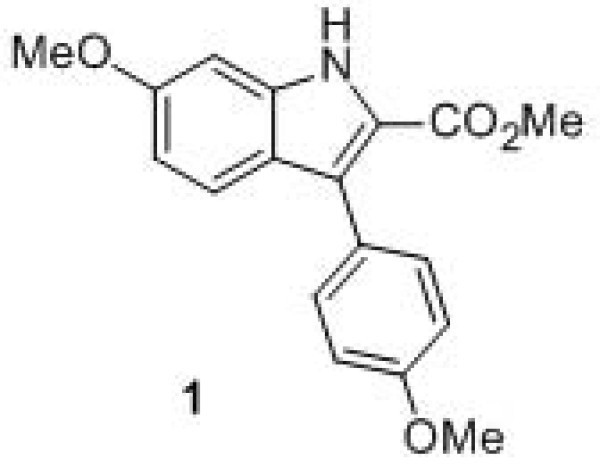
**Structure of methyl 6-methoxy-3-(4-methoxyphenyl)-1*H*-indole-2-carboxylate**.

In the present study, the antitumoral activity of the fluorescent indole derivative **1**, methyl 6-methoxy-3-(4-methoxyphenyl)-1*H*-indole-2-carboxylate (Figure 1), previously synthesized by us [10], was tested for the *in vitro *growth of three human tumor cell lines, showing very low GI_50 _values. Considering its promising utility as an antitumoral drug, compound **1 **was encapsulated in different nanoliposome formulations and the mean size, size distribution, zeta potential, and stability were evaluated, keeping in mind future drug delivery applications using this compound as an anticancer drug.

The intrinsic fluorescence of compound **1 **was used to obtain relevant information about its location in nanoliposomes and its diffusion across the membrane in dialysis experiments. For the latter, Förster resonance energy transfer (FRET) between compound **1 **(energy donor) and nitrobenzoxadiazole (NBD)-labelled lipids in different positions (at head group or fatty acid), acting as energy acceptor, was used to monitor compound behavior, as this photophysical process strongly depends on the donor-acceptor distance [11]. These studies are important, not only to evaluate the best liposome formulations to encapsulate this promising antitumoral agent, but also to confirm the possibility of compound **1 **to permeate the lipid bilayer (cell membrane model).

## Experimental

### Nanoliposome preparation

Dipalmitoyl phosphatidylcholine (DPPC), egg yolk phosphatidylcholine (Egg-PC), dipalmitoyl phosphatidylglycerol (DPPG), Ch, and dihexadecyl phosphate (DCP) were obtained from Sigma-Aldrich (St. Louis, MI, USA). Distearoyl phosphatidylcholine (DSPC) and distearoyl phosphatidylethanolamine-*N*-[methoxy(polyethylene glycol)-2000] (ammonium salt) (DSPE-PEG) were purchased from Avanti Polar Lipids (Alabaster, AL, USA). Fluorescent-labelled lipids *N*-(7-nitrobenz-2-oxa-1,3-diazol-4-yl)-1,2-dihexadecanoyl-*sn*-glycero-3-phosphoethanolamine (triethylammonium salt) (NBD-PE), 2-(6-(7-nitrobenz-2-oxa-1,3-diazol-4-yl)amino)hexanoyl-1-hexadecanoyl-*sn*-glycero-3-phosphocholine (NBD-C_6_-HPC), and 2-(12-(7-nitrobenz-2-oxa-1,3-diazol-4-yl)amino)dodecanoyl-1-hexadecanoyl-*sn*-glycero-3-phosphocholine (NBD-C_12_-HPC) were obtained from Invitrogen (Carlsbad, CA, USA).

Nanoliposomes were prepared by injection of an ethanolic solution of lipids/compound **1 **mixture in an aqueous buffer solution under vigorous stirring, above the lipid melting transition temperature (*ca*. 41°C for DPPC [12] and 39.6°C for DPPG [13]), followed by three extrusion cycles through 100 nm polycarbonate membranes. The final lipid concentration was 1 mM, with a compound/lipid molar ratio of 1:333.

### Encapsulation efficiency (percent)

The encapsulation efficiency (EE) was determined through fluorescence emission measurements. After preparation, liposomes were subjected to centrifugation in an Eppendorf 5804 R centrifuge (Hamburg, Germany) at 11,000 rpm for 60 min. The supernatant was pipetted out, and its fluorescence was measured, allowing to calculate the compound concentration using a calibration curve previously obtained. The encapsulation efficiency of compound **1 **was determined using the following equation:

### DLS and zeta potential measurements

The liposomes' mean diameter, size distribution (polydispersity index), and zeta potential were measured with dynamic light scattering (DLS) NANO ZS Malvern Zetasizer equipment (Worcestershire, UK), at 25°C, using a He-Ne laser of 633 nm and a detector angle of 173°. Five independent measurements were performed for each sample. Malvern dispersion technology software (DTS) (Worcestershire, UK) was used with multiple narrow mode (high-resolution) data processing, and mean size (nanometer), and error values were considered.

### Dialysis

Permeability studies of compound **1 **between DPPC/DPPG mixed liposomes (donor liposomes) and NBD-labelled DPPC/DPPG liposomes (acceptor liposomes) were performed using two different sizes of dialysis membranes (6 to 8 KDa and 12 to 14 KDa). Three fluorescent NBD-labelled lipids were used, either labelled at head group (NBD-PE) or labelled at fatty acid (NBD-C_6_-HPC and NBD-C_12_-HPC). The experiments were carried out using a reusable 96-well micro-equilibrium dialysis device HTC 96 (Gales Ferry, CT, USA) and left in an incubator at 25°C (80 rpm) for 36 h.

### Spectroscopic measurements

Fluorescence measurements were obtained in a Fluorolog 3 spectrofluorimeter (HORIBA Scientific, Kyoto, Japan), equipped with double monochromators in both excitation and emission and a temperature controlled cuvette holder. Fluorescence spectra were corrected for the instrumental response of the system. Nanoliposomes containing only the fluorescent compound **1 **(energy donor) served as negative (no FRET) control. The percentage of energy transfer, ET (percent), was calculated from the fluorescence emission intensities,

where *I_DA _*is the donor emission intensity after the dialysis experiment in NBD-labelled lipid/DPPC/DPPG liposomes,  is the initial donor emission intensity in DPPC/DPPG liposomes and  is the final donor emission intensity in DPPC/DPPG liposomes.

### Biological activity

Fetal bovine serum, L-glutamine, phosphate-buffered saline, trypsin, and RPMI-1640 medium were purchased from Invitrogen (Carlsbad, CA, USA). Acetic acid, dimethyl sulfoxide (DMSO), doxorubicin, penicillin, streptomycin, ethylenediaminetetraacetic acid, sulforhodamine B, and trypan blue were from Sigma-Aldrich (St. Louis, MI, USA). A stock solution of 1 was prepared in DMSO and was kept at -70°C. Appropriate dilutions of the compound were freshly prepared in the test medium just prior to the assays. The vehicle solvent had no influence on the growth of the cell lines. Human tumor cell lines MCF-7 (breast adenocarcinoma), NCI-H460 (non-small cell lung cancer), and A375-C5 (melanoma) were tested. MCF-7 and A375-C5 were obtained from the European Collection of Cell Cultures (Salisbury, UK), and NCI-H460 was kindly provided by National Cancer Institute (NCI) (Bethesda, MD, USA). The procedure followed was described elsewhere [14]. The *in vitro *effect on the growth of human tumor cell lines was evaluated according to the procedure adopted by the NCI in their "*In vitro *Anticancer Drug Discovery Screen," using the protein-binding dye sulforhodamine B to assess cell growth [15,16]. Doxorubicin was tested following the same protocol and was used as positive control.

## Results and discussion

### Antitumoral evaluation

The *in vitro *growth inhibitory activity of compound **1 **was evaluated using three human tumor cell lines, breast adenocarcinoma (MCF-7), non-small cell lung cancer (NCI-H460), and a melanoma cell line (A375-C5), after 48 h of continuous exposure to compound **1**. Results given in concentrations that were able to cause 50% of cell growth inhibition (GI_50_) are summarized in Table 1. It can be observed that compound **1 **inhibited the growth of the three tumor cell lines with very low GI_50 _values. These inhibitory concentrations are significantly lower than those obtained with other potential antitumoral compounds recently tested [17-19], some of them also containing the indole nucleus [17-21], and point to a promising utility of this compound as an antitumoral agent. Doxorubicin, used as positive control, presents a very high cytotoxicity because the planar aromatic moiety efficiently intercalates into DNA base pairs, while the six-membered daunosamine sugar binds to the minor groove, interacting with flanking base pairs adjacent to the intercalation site [22]. Nevertheless, doxorubicin presents also a high toxicity for the human body, and the search for other antitumoral compounds, even less active but also less toxic, is still an active domain of interest.

**Table 1:** Values of compound 1 concentration needed for 50% of cell growth inhibition (GI_50_)

	GI_50 _(μM)		
	MCF-7	NCI-H460	A375-C5
1	0.37 ± 0.02	0.33 ± 0.03	0.25 ± 0.02

Results represent means ± SEM of three independent experiments performed in duplicate. Doxorubicin was used as positive control (GI_50_: MCF-7 = 43.3 ± 2.6 nM; NCI-H460 = 35.6 ± 1.6 nM; and A375-C5 = 130.2 ± 10.1 nM).

### Nanoliposomes characterization

Selected liposome formulations [23-25] with encapsulated compound **1 **were prepared. All the formulations have mean hydrodynamic diameters lower than 120 nm, generally low polydispersity and very good encapsulation efficiency (Table 2). Pegylation of nanoliposomes surface with DSPE-PEG generally leads to the increase of the hydrodynamic diameter that, however, remains close to 100 nm. The mean diameter of the Egg-PC/DCP/Ch (7:2:1) liposome is considerably smaller than the others (Table 2), but with a higher polydispersity index. Formulations including egg phosphatidylcholine show a tendency to a lower particle size. All the different nanoliposomes prepared are generally monodisperse and stable after 2 weeks, with no evidence of aggregation (Table 2).

**Table 2:** Hydrodynamic diameter, polydispersity, zeta potential, and encapsulation efficiency of several drug-loaded liposomes

Drug-loaded liposomes	Hydrodynamic diameter (nm) (mean ± SD)	Polydispersity (mean ± SD)	Zeta potential (mV) (mean ± SD)	Encapsulation efficiency
DPPC/Ch/DSPE-PEG (5:5:1)	115.4 ± 0.5	0.15 ± 0.01	-30 ± 1	97%
1 week after	116 ± 2	0.15 ± 0.01		
2 weeks after	116.0 ± 0.8	0.15 ± 0.01		
DSPC/Ch/DSPE-PEG (5:5:1)	120 ± 2	0.19 ± 0.01	-27 ± 4	96%
Egg-PC/Ch/DSPE-PEG (5:5:1)	104.3 ± 0.6	0.25 ± 0.01	-19 ± 2	99%
Egg-PC/DCP/Ch (7:2:1)	79.3 ± 0.8	0.37 ± 0.01	-39 ± 3	98%
Egg-PC/Ch/DPPG (6.25:3:0.75)	103.5 ± 0.9	0.12 ± 0.01	-52 ± 6	98%
2 weeks after	95.4 ± 0.5	0.14 ± 0.01		
Egg-PC/DPPG/DSPE-PEG (5:5:1)	104 ± 3	0.27 ± 0.01	-43 ± 3	99%

Standard deviations were calculated from the mean of the data of a series of five experiments conducted using the same parameters.

Zeta potential measurements were used to evaluate the relationship between surface charge and stability. All the nanoliposome formulations have negative zeta potential (Table 2). The higher colloidal stability was obtained for Egg-PC/Ch/DPPG (6.25:3:0.75) formulation (ζ value more negative), while the lower stability (higher aggregation tendency) is observed for Egg-PC/Ch/DSPE-PEG (5:5:1) liposomes, which exhibit a ζ-potential value clearly less negative than -30 mV.

### Dialysis

Previous fluorescence experiments showed the possibility of FRET between the excited compound **1 **and the widely used fluorescence probe nitrobenzoxadiazole, NBD. The FRET mechanism involves a donor fluorophore in an excited electronic state (here compound **1**), which may transfer its excitation energy to a nearby acceptor chromophore (NBD) in a nonradiative way through long-range dipole-dipole interactions. Because the range over which the energy transfer can occur is limited to approximately 100 Å and the efficiency of transfer is extremely sensitive to the donor-acceptor separation distance, resonance energy transfer measurements can be a valuable tool for probing molecular interactions [11].

**Figure 2:**
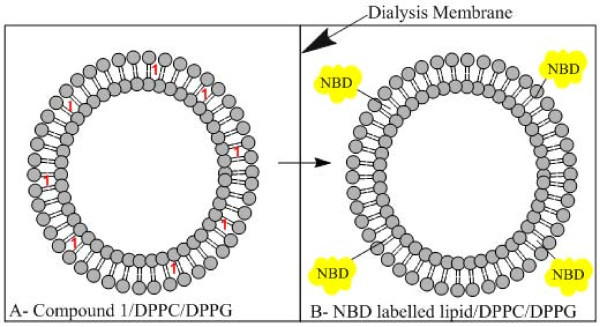
**Schematic dialysis experiment from DPPC/DPPG liposomes to NBD-labelled lipid/DPPC/DPPG liposomes**.

Taking advantage of the possibility of FRET from the excited compound **1 **(donor) to the nitrobenzoxadiazole moiety, the diffusion of compound **1 **in dialysis experiments was monitored using this photophysical process. Two different dialysis membranes (6 to 8 KDa or 12 to 14 KDa) were tested. The experiments were carried out at 25°C for 36 h and are schematically illustrated in Figure 2. DPPC/DPPG (1:1) liposomes with encapsulated compound **1 **(donor liposomes) were placed at one side of the dialysis membrane (Figure 2, left), while NBD-labelled lipid/DPPC/DPPG liposomes without compound (acceptor liposomes) are placed at the other side (Figure 2, right). After the experiment (36 h), the occurrence of energy transfer (FRET) from compound **1 **to NBD, detected in the solution located at the right side, is a proof of compound diffusion from the donor liposomes, passing across the dialysis membrane and incorporation in the membrane of the acceptor liposomes. The phospholipids DPPC and DPPG are the main components of biological membranes and are both in the gel phase at room temperature. This fact is expected to restrain the diffusion of compound **1 **and, therefore, if the compound diffuses through the dialysis membrane in this situation, this will be even easier with the lipids that are in the fluid phase.

**Figure 3:**
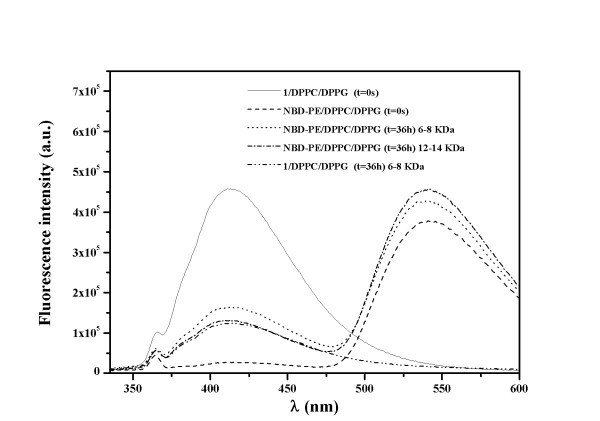
**Fluorescence spectra of compound 1 in DPPC/DPPG liposomes and NBD-PE labelled DPPC/DPPG liposomes before and after dialysis**.

The NBD-labelled lipids were either labelled at head group (NBD-PE), at position 6 of the fatty acid chain (NBD-C_6_-HPC) or at position 12 of the fatty acid chain (NBD-C_12_-HPC). Figure 3 displays (as examples) the emission spectra of compound **1 **in DPPC/DPPG donor liposomes and of the NBD-PE/DPPC/DPPG acceptor nanoliposomes, before (*t *= 0 s) and after (*t *= 36 h) diffusion of compound **1 **through the two dialysis membranes used in the study. After the dialysis assay, the fluorescence of compound **1 **in the donor liposomes is notably reduced (Figure 3), and its emission can be detected in the acceptor liposomes solution, showing the diffusion of compound **1 **through the dialysis membrane. Besides, due to the energy transfer from compound **1 **to NBD, the fluorescence intensity of the latter notably increases (Figure 3). The effect is stronger for the membrane of 12 to 14 KDa.

**Figure 4:**
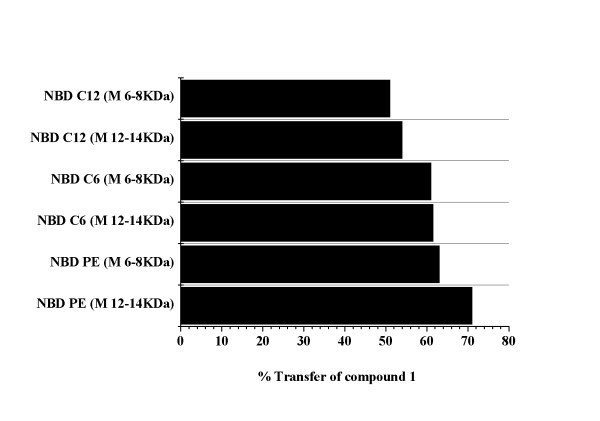
**Percentage of drug transfer in dialysis between DPPC/DPPG liposomes and NBD-labelled lipid/DPPC/DPPG liposomes**.

The percentage of energy transfer from compound **1 **to NBD is higher when the acceptor nanoliposomes are labelled with NBD-PE (NBD linked at lipid head group) (Figure 4). In this case, it can be observed that energy transfer is higher for the 12- to 14-KDa dialysis membrane. It can also be concluded that, after 36 h of dialysis, compound **1 **is located mainly near the polar head groups of the phospholipids in the acceptor nanoliposomes, as energy transfer to NBD is less efficient when this energy acceptor is located deeper in the lipid chain (NBD-C_12 _or NBD-C_6_) (Figure 4).

## Conclusions

The fluorescent methyl 6-methoxy-3-(4-methoxyphenyl)-1*H*-indole-2-carboxylate (**1**) exhibits excellent antitumoral properties, with very low GI_50 _values in the three human tumor cell lines tested. Several nanoliposome formulations containing the fluorescent drug were successfully prepared by an injection/extrusion combined method, with particle sizes lower than 120 nm, low polydispersity index, and good stability after 2 weeks. The Egg-PC/Ch/DPPG (6.25:3:0.75) and Egg-PC/DPPG/DSPE-PEG (5:5:1) showed to be the best formulations for encapsulation of this compound, considering their low hydrodynamic diameter, high negative zeta potential, and very high encapsulation efficiency. Dialysis experiments allowed to follow compound diffusion from DPPC/DPPG donor liposomes to NBD-labelled lipid/DPPC/DPPG acceptor liposomes, through dialysis membranes of 6 to 8 KDa and 12 to 14 KDa. These results may be important for future drug delivery applications using nanoliposomes for the encapsulation and transport of this promising antitumoral compound. Further developments of the present study will involve assays of liposome cell internalization and mechanism of action, keeping in mind the application of this compound as an antitumoral drug.

## Abbreviations

A375-C5: melanoma cell line; Ch: cholesterol; DCP: dihexadecyl phosphate; DLS: dynamic light scattering; DPPC: dipalmitoyl phosphatidylcholine; DPPG: dipalmitoyl phosphatidylglycerol; DSPC: distearoyl phosphatidylcholine; DSPE:PEG: 1,2-distearoyl-*sn*-glycero-3-phosphoethanolamine-*N*-[methoxy(polyethylene glycol)-2000]; DTS: dispersion technology software; Egg-PC: egg yolk phosphatidylcholine; FRET: Förster resonance energy transfer; MCF-7: breast adenocarcinoma cell line; NBD-C_6_-HPC: 2-(6-(7-nitrobenz-2-oxa-1,3-diazol-4-yl)amino)hexanoyl-1-hexadecanoyl-*sn*-glycero-3-phosphocholine; NBD-C_12_-HPC: 2-(12-(7-nitrobenz-2-oxa-1,3-diazol-4-yl)amino)dodecanoyl-1-hexadecanoyl-*sn*-glycero-3-phosphocholine; NBD-PE: *N*-(7-nitrobenz-2-oxa-1,3-diazol-4-yl)-1,2-dihexadecanoyl-*sn*-glycero-3-phosphoethanolamine; NCI-H460: non-small cell lung cancer line.

## Competing interests

The authors declare that they have no competing interests.

## Authors' contributions

ASA and EMSC conceived the study, were responsible for the interpretation of results, and drafted the manuscript. ASA carried out the liposome preparation, the DLS and zeta potential measurements and dialysis experiments in liposomes. M-JRPQ and PMF supervised the organic synthesis and compound characterization and participated in the draft of the manuscript. LAVS was responsible for the antitumoral evaluation of the compound. EP supervised the studies of biological activity. All authors read and approved the final manuscript.
